# ALDH1A3 Is the Key Isoform That Contributes to Aldehyde Dehydrogenase Activity and Affects *in Vitro* Proliferation in Cardiac Atrial Appendage Progenitor Cells

**DOI:** 10.3389/fcvm.2018.00090

**Published:** 2018-07-24

**Authors:** Stefania Puttini, Isabelle Plaisance, Lucio Barile, Elisabetta Cervio, Giuseppina Milano, Paola Marcato, Thierry Pedrazzini, Giuseppe Vassalli

**Affiliations:** ^1^Cardiovascular Department, CHUV University Hospital, Lausanne, Switzerland; ^2^Cardiocentro Ticino Foundation and Swiss Institute for Regenerative Medicine, Lugano, Switzerland; ^3^Departments of Pathology, Microbiology and Immunology, Dalhousie University, Halifax, NS, Canada

**Keywords:** aldehyde dehydrogenase, ALDH1A3, cardiac progenitor cell, stem cell, heart

## Abstract

High aldehyde dehydrogenase (ALDH^hi^) activity has been reported in normal and cancer stem cells. We and others have shown previously that human ALDH^hi^ cardiac atrial appendage cells are enriched with stem/progenitor cells. The role of ALDH in these cells is poorly understood but it may come down to the specific ALDH isoform(s) expressed. This study aimed to compare ALDH^hi^ and ALDH^lo^ atrial cells and to identify the isoform(s) that contribute to ALDH activity, and their functional role.

**Methods and Results:** Cells were isolated from atrial appendage specimens from patients with ischemic and/or valvular heart disease undergoing heart surgery. ALDH^hi^ activity assessed with the Aldefluor reagent coincided with primitive surface marker expression (CD34^+^). Depending on their ALDH activity, RT-PCR analysis of ALDH^hi^ and ALDH^lo^ cells demonstrated a differential pattern of pluripotency genes (Oct 4, Nanog) and genes for more established cardiac lineages (Nkx2.5, Tbx5, Mef2c, GATA4). ALDH^hi^ cells, but not ALDH^lo^ cells, formed clones and were culture-expanded. When cultured under cardiac differentiation conditions, ALDH^hi^ cells gave rise to a higher number of cardiomyocytes compared with ALDH^lo^ cells. Among 19 ALDH isoforms known in human, ALDH1A3 was most highly expressed in ALDH^hi^ atrial cells. Knocking down ALDH1A3, but not ALDH1A1, ALDH1A2, ALDH2, ALDH4A1, or ALDH8A1 using siRNA decreased ALDH activity and cell proliferation in ALDH^hi^ cells. Conversely, overexpressing ALDH1A3 with a retroviral vector increased proliferation in ALDH^lo^ cells.

**Conclusions:** ALDH1A3 is the key isoform responsible for ALDH activity in ALDH^hi^ atrial appendage cells, which have a propensity to differentiate into cardiomyocytes. ALDH1A3 affects *in vitro* proliferation of these cells.

## Introduction

In many studies, putative cardiac-resident stem and progenitor cells were isolated based on the expression of hematopoietic stem cell (HSC) markers, such as CD34, stem cell antigen-1 (Sca-1), and *c*-kit/CD117 (1–3). However, no single cell surface marker definitely identifies a specific entity of cardiac stem cells (4). Therefore, “functional” stem cell markers have been intensively searched for. It has been proposed that, irrespective of their lineage origin, stem cells may respond in similar ways to regulate self-renewal and differentiation, and that putative “stemness” genes may serve as universal stem cell markers (5). A widely used universal stem cell marker is aldehyde dehydrogenase (ALDH) (6–8). In tunicates, few pluripotential cells with high ALDH enzymatic activity (ALDH^hi^) were able to give rise to all somatic and reproductive cell lineages (9).

ALDH is a generic designation for a superfamily of NAD-dependent enzymes that catalyze the oxidation of aldehydes to acids. The ALDH family includes 19 members in human, which share some amino acid sequence similarity and certain functions while differing in intracellular localization, tissue distribution, and substrate specificity. ALDHs are localized in the cytoplasm, mitochondria or nucleus and participate in many biological processes including the detoxification of exogenous aldehydes and the metabolism of vitamin A, alcohol and reactive oxygen species (ROS) (7, 10). ALDH1A1 is a detoxifying enzyme responsible for oxidizing aldehydes to carboxylic acids (11). High-level ALDH1A1 expression regulates self-renewal and differentiation in HSCs and neural stem cells (12–14). ALDH1A1 has been implicated in chemotherapeutic resistance of HSCs via metabolization and detoxification of chemotherapeutics like cyclophosphamide (15). ALDH1A is the rate-limiting factor in the production of retinoic acid (RA) via oxidation of *all-trans* retinal and 9-cis-retinal (16–18). RA activates nuclear RA receptors (RARs) that control the transcription of genes with RA response elements (RAREs) in their promoters, thereby regulating stem cell functions (13, 19). Elevated activity of additional ALDH isoforms, namely ALDH1A2, ALDH1A3, ALDH1A7, ALDH2^*^2, ALDH3A1, ALDH4A1, ALDH5A1, ALDH6, and ALDH9A1, has been observed in normal and cancer stem cells (10, 20–25). It has been proposed that the role of ALDH as a stem cell marker may come down to the specific isoform(s) expressed (20). Thus, ALDH not only may be considered a stem cell marker, but also may well play functional roles in terms of self-renewal, differentiation, and/or expansion. It should be noted, however, that currently available commercial assays identifying ALDH^hi^ cells as those actively metabolizing BODIPY-aminoacetaldehyde (Aldefluor®) (26) do not distinguish the specific ALDH isoforms (8).

In human, ALDH expression by HSCs has been evaluated as a predictor of hematopoietic recovery after peripheral stem cell mobilization (27) and a biomarker for umbilical cord blood potency (28). Both bone marrow and cord blood-derived ALDH^hi^ cells have shown therapeutic potential in limb ischemia (29) and myocardial infarction models (30). In clinical trials, autologous bone marrow-derived ALDH^hi^ cells did not improve functional or magnetic resonance outcomes in patients with peripheral artery disease (31). More encouraging results were reported in patients with ischemic heart failure (32). We were the first to isolate cardiac atrial appendage-derived progenitor cells based on ALDH activity (33, 34). Koninckx et al. (35) then reported that human ALDH^hi^ cardiac atrial appendage stem cells (CASC) gave rise to cardiac cells and improved cardiac function upon injection into infarcted pig hearts. However, this study did not compare ALDH^hi^ and ALDH^lo^ cells nor did it define the specific ALDH isoform(s) expressed and their functional roles.

The present study aimed to compare human ALDH^hi^ and ALDH^lo^ atrial appendage cells both phenotypically and functionally, and to identify the specific ALDH isoform(s) expressed. ALDH1A3 was found to be the key isoform responsible for Aldefluor positivity in ALDH^hi^ cells. Gain- and loss-of-function experiments revealed a role for ALDH1A3 in cell proliferation.

## Materials and methods

### Cell isolation and flow cytometric analysis

Human right atrial appendage specimens were obtained from male and female patients (29–91 years old) who underwent cardiac surgery for ischemic and/or valvular heart disease through donation. The protocol received authorization from the University Hospital Ethics Committee and the Cantonal Ethics Committee Ethics Committee of Canton Vaud, Switzerland on research involving humans. Informed, written consent was obtained from the participants. In 3 patients (76–86 years old) who underwent left ventricular (LV) assist device implantation, tissue specimens were obtained from the LV apex. Immediately after their procurement, tissue specimens were kept on ice, minced, and digested in a buffer containing 0.45 mg/ml collagenase from Clostridium histolyticum and 0.1 mg/ml proteinase bacterial Type XXIV (both from Sigma Aldrich, St. Louis, MO, USA). Four rounds of enzymatic digestion were used. Freshly isolated cells were immediately reacted with Aldefluor (Stem Cell Technologies, Vancouver, BC, Canada) to identify ALDH^hi^ cells. Briefly, 2 × 10^6^ cells/mL were suspended in Aldefluor assay buffer containing the ALDH substrate BODIPYaminoacetaldehyde and incubated at 37°C for 45 min. For each sample, cell aliquots were incubated with or without 50 mM diethylaminobenzaldehyde (DEAB), an ALDH-specific inhibitor (36), and analyzed on a Gallios flow cytometer (Beckman Coulter, Indianapolis, IN, USA). The threshold used for the ALDH^hi^ gate was 2.0% of DEAB-treated control cells. Dead cells and cells in the early-mid apoptosis were identified using DAPI and Annexin V apoptosis detection kit-APC (eBioscience; Thermo Fisher Scientific, Waltham, MA, USA), respectively. For flow cytometric analyses of surface marker expression, 1 × 10^5^ cells/tube were incubated with Aldefluor and subsequently stained with marker-specific antibodies (Supplementary Table 1) for 30 min in the dark. Fluorescence-activated cell sorting (FACS) based on ALDH activity was performed on a MoFloAstrios cell sorter (Beckman Coulter). To prevent cross-contamination between ALDH^hi^ and ALDH^hi^ cells, sorting gates of these 2 populations were set up at least one log apart. The purity of sorted populations was reanalyzed using ALDH^hi^ and ALDH^lo^ cells and was shown to be greater than 95%. For expansion, cells were plated in gelatin-coated 6-well plates and cultured in expansion medium (3:1 DMEM 1g/l glucose/Medium 199 [Invitrogen, Carlsbad, CA, USA] supplemented with 10% horse serum [Serotec; BioRad Labs., Hercules, CA, USA], 5% fetal calf serum (FCS) [Serotec], 100 U/ml penicillin [Invitrogen], and 100 mg/ml streptomycin [Invitrogen]). Cells that remained in suspension after 24 h in culture were discarded, and adherent cells were expanded. In a subset of experiments, atrial appendage specimens were not dissociated using enzymatic techniques, but cultured *ex vivo* as primary tissue explants (37). Briefly, atrial tissue explants were cut into small pieces (≈1 mg) that were placed in gelatin-coated culture dishes (Corning; Thermo Fisher Scientific) and cultured in IMDM supplemented with 20% FCS, 100 U/ml penicillin and 100 mg/ml streptomycin. After 10 days, the small pieces of tissue were removed from culture dishes and the cell populations that had migrated out of them (outgrowth cells) were expanded.

### Immunocytochemistry

Immunocytochemical staining was performed on cells cultured in gelatin-coated wells using the antibodies listed in Supplementary Table 2. Stained cells were mounted with Vectashield mounting medium containing DAPI (Vector Labs, Burlingame, CA, USA). Images were captured using an Axiovision (Carl Zeiss Jena, Germany) fluorescence microscope and a Nikon LSR Eclipse TE2000S inverted fluorescence microscope (Nikon, Tokyo, Japan).

### Clonogenicity and cell proliferation assays

To assess clonogenicity, atrial cells were seeded at limit dilution and single cells were identified under a light microscope. Clones derived from single cells were counted at later time points. To assess cell growth, cell numbers were counted at different time points after plating. The effect of DEAB on proliferation was assessed in outgrowth cells. Cell proliferation was measured using Click-iT® EdU Alexa Fluor® 488 imaging kit (Invitrogen), as per manufacturer's instructions. Briefly, cells were incubated with EdU (10 μM final concentration) for 2 h in expansion medium, fixed with paraformaldehyde 2%, permeabilized with 0.3% Triton, incubated with Alexafluor azide for 30 min in the dark, and mounted with Vectashield. EdU^+^ cells and total cells were counted under a fluorescence microscope, and percentages of EdU^+^ cells were calculated. Cell density was expressed using a microscopic score (range: 0–4) assigned by two independent investigators blind to experimental conditions. RA levels in cells were measured using Human Retinoic Acid ELISA Kit (MBS705877-96; ANAWA Biotrend, Wangen, Switzerland). The ability of the RA receptor agonist all*-trans* RA to replace ALDH in promoting cell growth was tested. Varying concentrations of all*-trans* RA (Sigma) were added to explant outgrowths cultured in the presence of 100 μM DEAB, and cell density was assessed under a light microscope.

### RNA isolation, reverse transcription, and real-time PCR

Total RNA was extracted using RNeasy isolation kit (Qiagen N V, Hilden, Germany). Reverse transcription was performed with QuantiTect Whole Transcriptome Kit (Qiagen) for RNA from freshly isolated cells, and with Verso cDNA Synthesis Kit (Thermo Fisher) for RNA from culture-expanded cells. For expression analyses of cardiac transcription factors, pluripotency-associated genes and cardiac differentiation markers, cDNA was used as a template for real-time PCR or relative quantitative PCR (qPCR) using specific probes and the ABI Prism 7500 Fast Real-Time PCR System (Applied Biosystems; Thermo Fisher). For expression analyses of ALDH isoforms, Fast SYBR®Green Master Mix (Life Technologies; Thermo Fisher) with gene-specific primers (Thermo Fisher; Supplementary Table 3) were used. Each reaction was carried out in triplicate. To calculate the relative expression levels, we used the 2^−ΔΔCt^-method. RPL27 mRNA was used as an endogenous control. Experiments linked to differentiation were done with Taqman probes using β2–microglobuline as a reference gene.

### Western blotting

Western blotting was performed on ALDH^hi^ and ALDH^lo^ sorted cells using rabbit anti-human ALDH1A3 antibody (Ab 129815, Abcam, Cambridge, UK).

### Cardiomyogenic differentiation

Atrial cells at culture-passage 2 were reacted with Aldefluor. ALDH^hi^ and ALDH^lo^ populations were sorted. Two protocols were used to induce cardiac differentiation. Using the first protocol (no NOTCH modulation), differentiation was induced by switching the cells to MEM alpha (Invitrogen) containing 2% horse serum, 1 mmol/l dexamethasone (Sigma-Aldrich), 50 mg/ml ascorbic acid (Sigma-Aldrich), 10 mmol/l b-glycerophosphate (Sigma-Aldrich), 100 U/ml penicillin, and 100 mg/ml streptomycin (differentiation medium). Because cells were spontaneously differentiating into smooth muscle cells using this protocol, a recently developed protocol involving sequential NOTCH activation and inhibition was applied (38). Cells were cultured on immobilized Delta-like1 (DLL1) ligand (R&D Systems, Minneapolis, MN, USA) during 24 h. After cell re-suspension, NOTCH pathway was inhibited by the inhibitor of gamma secretase, DAPT (Sigma-Aldrich). Cells were cultured under those conditions for 3 weeks in differentiation medium, and cells were then assessed for expression of cardiac-specific (Myh6, Myh7) and smooth muscle (SM)-specific (Myh11) genes using quantitative RT-PCR. Expression of cardiac-specific α-actinin and troponin I (cTnI), as well as SM myosin heavy chain (sm-MHC) was assessed immunocytochemically. Cells positive for α-actinin or cTnI, or both markers, and total cells were counted under the fluorescence microscope. Percentages of positive cells were calculated.

### siRNA silencing

Small interfering RNA (siRNA) was used to knockdown ALDH isoform expression in atrial cells. All siRNAs were obtained from Thermo Fisher. ALDH^hi^ sorted cells seeded on 6 well-plates (50.000 cells/well) were allowed to adhere overnight. Transfection was performed with Lipofectamine RNAiMAX (Invitrogen), as per manufacturer's instructions. siALDH1A3 was tested at different concentrations. In subsequent experiments, siRNA specific for ALDH isoforms 1A1, 1A2, 1A3, 2A, 4A1, 8A1 was used at the 10 nM concentration. Percentages of ALDH^hi^ cells were measured at 24 h post-transfection using the Aldefluor assay. EdU was added to cells at 24 h post-transfection, and EdU incorporation was assessed using Click-iT® EdU Alexa Fluor® 488 imaging kit.

### ALDH1A1 and ALDH1A3 overexpression

The retroviral vector pMSCVpuro with either ALDH1A1 or ALDH1A3 coding sequences inserted was described and validated previously (32). A retroviral vector with no insert was used to control for unspecific virus-related effects. ALDH^lo^ sorted cells were transduced with retroviral supernatants in 6-well plates (50,000 cells/well), as described previously (32). Twenty-four hours post-transduction, cells were passaged and seeded at ≈50% density. EdU was added to the cells 24 h later. Cell proliferation was analyzed using Click-iT® EdU Alexa Fluor® 488 imaging kit.

### Statistics

The data are presented as mean ± S.D. Student *t*-test was used to compare two normally distributed populations. The Mann-Whithney U test was used for nonparametric comparisons of two groups. Differences with probability values *p* < 0.05 were considered statistically significant.

## Results

### Identification of ALDH^hi^ cells

**Figure 1:**
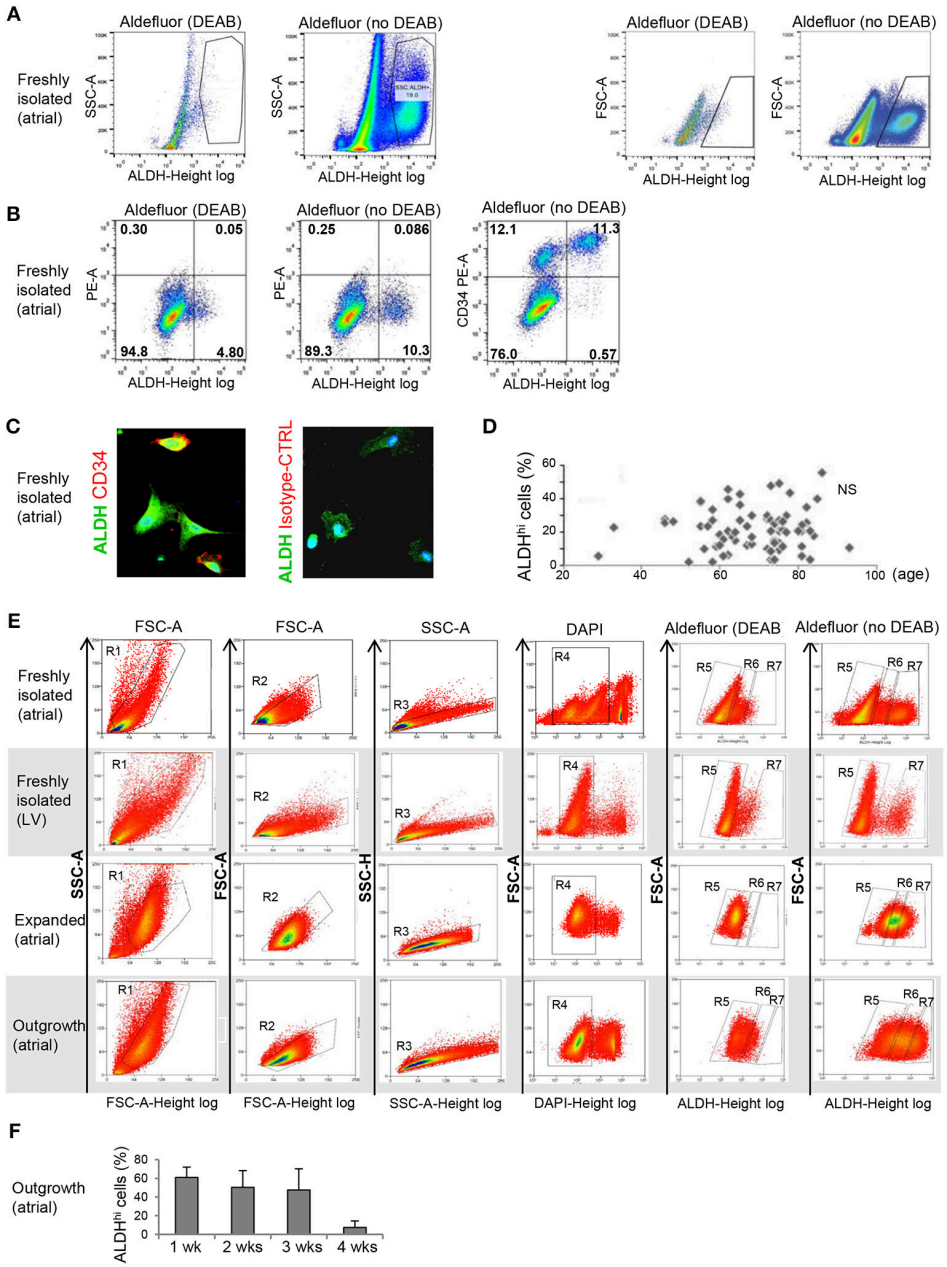
Flow cytometry analysis of ALDH^hi^ atrial cells. **(A)** Freshly isolated cells. ALDH^hi^ gating was established by inhibiting ALDH activity in Aldefluor-reacted cells with DEAB. Left panels: ALDH/side scatter (SSC-A) plots. Right panels: ALDH/forward scatter (FSC-A) plots. **(B)** Freshly isolated cells. Aldefluor activity and CD34 expression. **(C)** CD34 immunostaining (red); ALDH (green); staining with istotype-matched control antibody is shown. **(D)** ALDH^hi^ cells (%) did not correlate with age. **(E)** FACS plots of different populations (top to bottom: freshly isolated atrial cells, freshly isolated LV cells, expanded atrial cells, atrial outgrowth cells). Gating sequences are shown. R1-gated cells in FSC-A/SSC-A plots are gated for homogeneity (R2, R3) and DAPI exclusion (R4). R5 and R7 gates define ALDH^lo^ and ALDH^hi^ cells, respectively. To prevent cross-contamination between ALDH^hi^ and ALDH^hi^ cells, R5 and R7 gates were set one log apart (i.e., R6-gated cells were discarded). **(F)** Percentages of ALDH^hi^ outgrowth cells after 1–4 weeks of *ex vivo* tissue culture.

Freshly isolated, myocyte-depleted populations were isolated based on ALDH activity using the Aldefluor assay. ALDH^hi^ gating was established by inhibiting ALDH activity using DEAB (Figure 1A). Freshly isolated cells from right atrial appendages contained an average of 21.5 + 12.4% ALDH^hi^ cells (*n* = 86). In a few LV apex specimens available (*n* = 3), ALDH^hi^ cells averaged 12.3 ± 2.1% of total cells. This finding demonstrated that the LV apex also contains ALDH^hi^ cells; however, quantitative comparisons of atrial and LV cells were not performed due to the small LV sample size. A majority of ALDH^hi^ atrial cells (73.1 + 20.2%) expressed the primitive surface marker CD34 (Figure 1B), which was confirmed immunocytochemically (Figure 1C), but not CD45 (<5%). There was no significant correlation between ALDH^hi^ cell number and age (Figure 1D), gender, or ischemic vs. heart valve disease (data not shown). Because freshly isolated ALDH^lo^ cells could not be expanded *ex vivo*, we used ALDH^lo^ outgrowth cells (37) in a subset of experiments including direct comparisons with ALDH^hi^ cells. In addition, outgrowth cells are of translational interest, as they give rise to cardiosphere-derived cells that have been tested clinically in patients after myocardial infarction (39). Representative gating sequences from FACS experiments with different populations, namely freshly isolated atrial cells, freshly isolated LV cells, expanded atrial cells, and outgrowth cells are shown in Figure 1E. In freshly isolated cells, but not in expanded ones and outgrowth cells, ALDH^hi^ and ALDH^lo^ cells were visualized as discrete populations, and the Aldefluor signal was incompletely inhibited by DEAB. By contrast, the Aldefluor signal was fully suppressed by DEAB in both expanded and outgrowth cells. These results suggest that ALDH activity in freshly isolated cells may be higher than in expanded and outgrowth cells. The frequency of ALDH^hi^ cells within the outgrowth population was stable during 3 weeks but decreased thereafter (Figure 1F), suggesting that the pool of ALDH^hi^ cells within cultured tissue explants may be exhausted at late time points.

### Freshly isolated ALDH^hi^ cells express CD34 along with mesenchymal and endothelial markers

**Figure 2:**
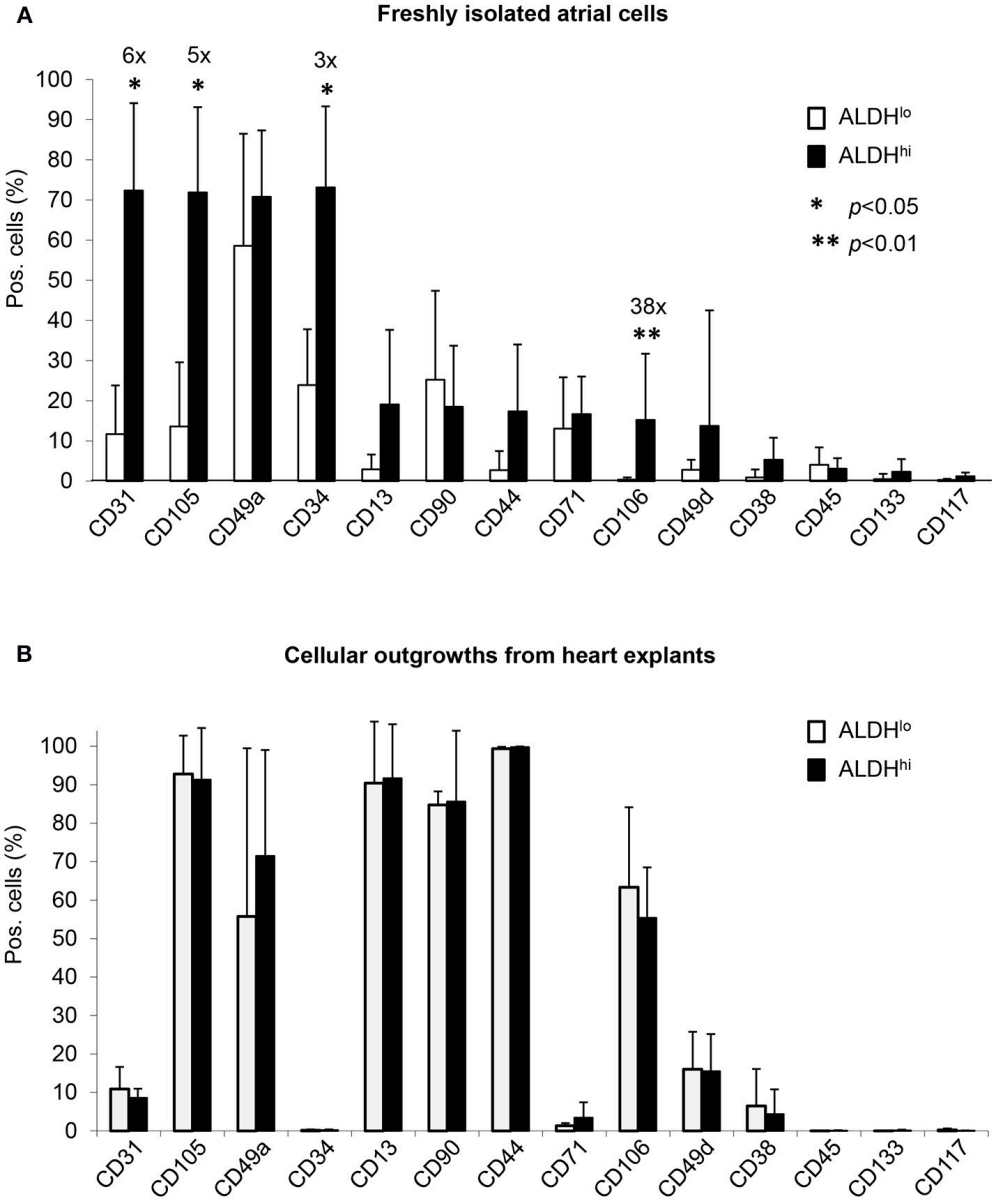
Flow cytometry analyses of cell-surface marker expression in ALDH^hi^ and ALDH^lo^ atrial cells. **(A)** Freshly isolated cells. Aldefluor-reacted cells were stained with antibodies to the indicated markers (see Supplementary Table 1). CD31^+^, CD105^+^, CD34^+^, and CD106^+^ cells (%) are significantly increased in ALDH^hi^ cells relative to ALDH^lo^ cells (fold-increases are indicated); *p < 0.05. **(B)** Outgrowth cells. ALDH^hi^ cells lack CD34 expression and exhibit a marker profile similar to that of ALDH^lo^ cells (*n* ≥ 5 per marker and group).

Differential surface marker expression in ALDH^hi^ and ALDH^lo^ atrial cells has not been analyzed previously. Freshly isolated ALDH^hi^ cells (Figure 2A) expressed the primitive marker CD34, platelet endothelial cell adhesion molecule-1 (PECAM-1)/CD31, and endoglin/CD105, a marker expressed by both endothelial and stromal progenitor cells. Thus, the ALDH^hi^ population appeared to include both mesenchymal progenitors and differentiating endothelial progenitors. The ALDH^hi^ population was enriched with cells expressing vascular cell adhesion molecule-1 (VCAM-1)/CD106 (38-fold increase). Small cell subsets expressed the common leukocyte antigen CD45 (<5%) and stem cell markers *c*-kit/CD117 (1.2 ± 0.9%) and prominin/CD133 (2.3 ± 3.1%). ALDH^hi^ outgrowth cells lacked CD34 expression. Their surface marker profile did not significantly differ from that of ALDH^lo^ cells (Figure 2B).

### Differential expression of pluripotency genes and markers for cardiac lineages

**Figure 3:**
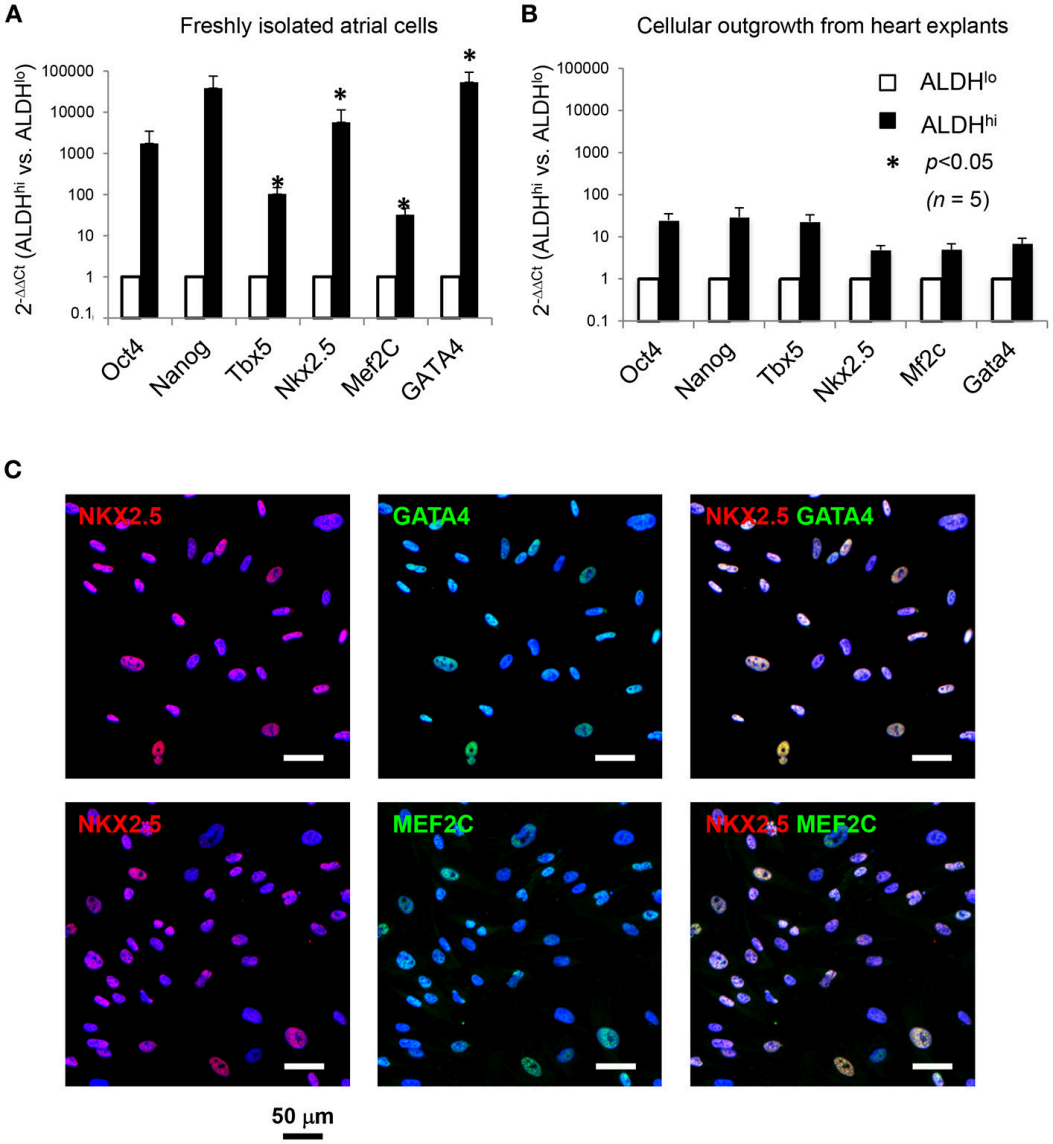
Expression of pluripotency-associated genes (Oct4, Nanog) and cardiac-specific transcription factors (Nkx2.5, Mef2c, GATA4, Tbx5). **(A)** Quantitative RT-PCR analyses of freshly isolated atrial cells (*n* = 5/group). **(B)** Outgrowth cells (*n* = 5/group). 2^−ΔΔCt^ values for ALDH^hi^ vs. ALDH^lo^ cells are shown. **(C)**. Freshly isolated ALDH^hi^ cells. Immunostaining for Nkx2.5 (red), GATA4 (green), and Mef2c (green); DAPI nuclear counterstaining (blue). Nkx2.5/GATA4 and Nkx2.5/Mef2c double stainings are shown.

We analyzed expression of pluripotency-associated genes and cardiac-specific markers as evidence of presence of primitive cells and cardiac progenitor cells, respectively, in atrial cell populations. Freshly isolated ALDH^hi^ cells expressed pluripotency-associated genes (Oct4, Nanog) and cardiac-specific transcription factors (Nkx2.5, Mef2c, GATA4, Tbx5) at higher levels (3–5 orders of magnitude) than did ALDH^lo^ cells (Figure 3A). Nkx2.5, GATA4, and Mef2c expression was confirmed immunocytochemically (Figure 3C). Similar albeit weaker differences (1–2 orders of magnitude) were found between ALDH^hi^ and ALDH^lo^ outgrowth cells (Figure 3B). These results suggest that the freshly isolated ALDH^hi^ population may be enriched with primitive cells and cells committed to a cardiac fate.

### Freshly isolated ALDH^hi^ cells are clonogenic and selectively expandable

**Figure 4:**
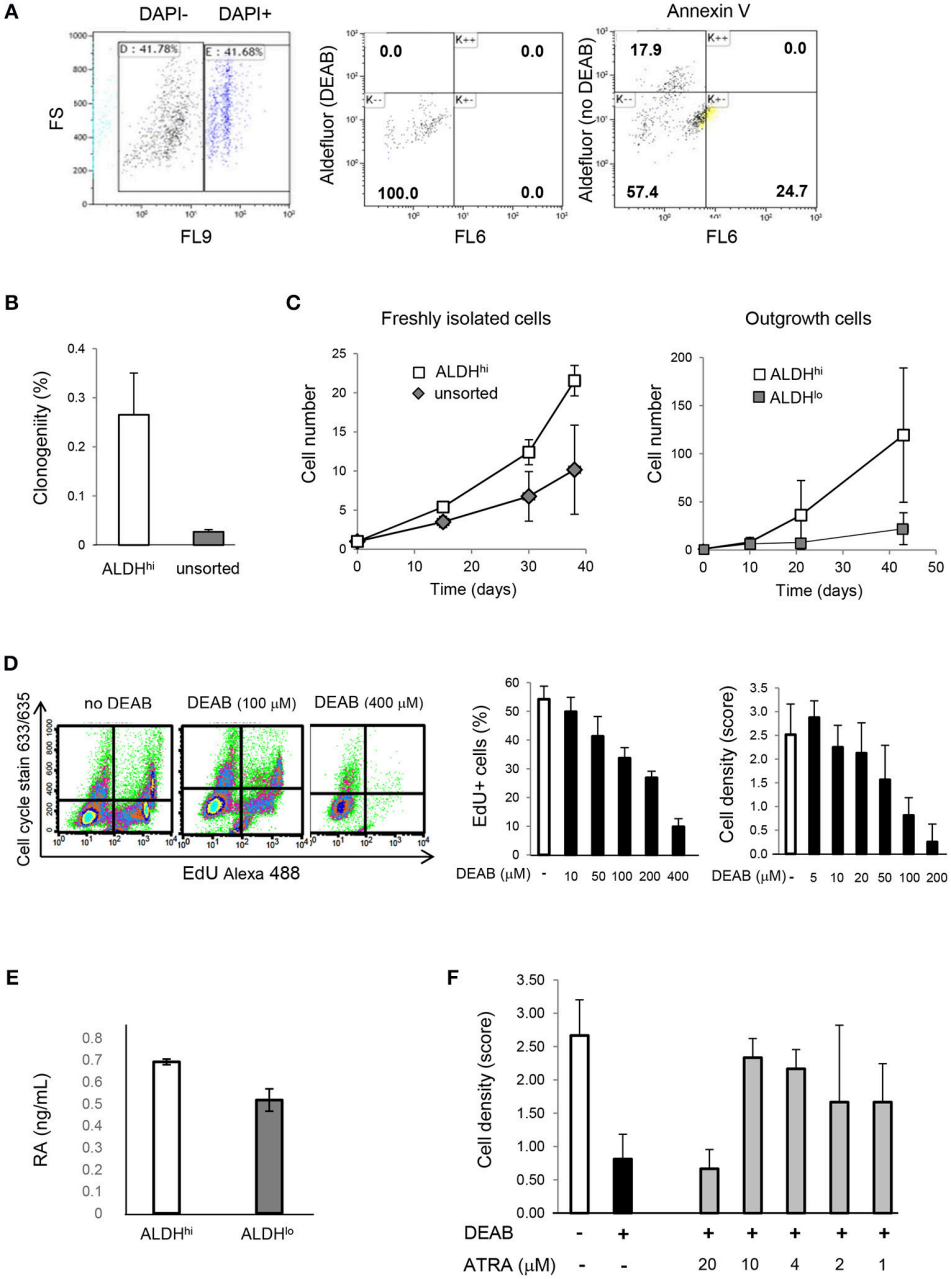
Cell viability, clonogenicity, cell growth, intracellular RA levels, effects of DEAB and all*-trans* RA. **(A)** Freshly isolated cells. Flow cytometry analysis of DAPI, Aldefluor (with and without DEAB), and Annexin V (right panel). Part of ALDH^lo^ cells are Annexin V-positive (K-+). **(B)** Freshly isolated ALDH^hi^ cells and unsorted cells. Clonogenic analysis (*n* = 3/group). **(C)** *In vitro* growth kinetics. Left panel: freshly isolated cells; right panel: outgrowth cells (*n* = 3/group in both panels). **(D)** Dose-dependent effects of DEAB on EdU incorporation and cell density. **(E)** RA concentrations in ALDH^hi^ and ALDH^lo^ cells measured by ELISA. **(F)** Dose-dependent effects of all*-trans* RA (ATRA) on cell density in the presence of DEAB (100 μM).

Freshly isolated ALDH^hi^ cells selectively formed clones and could be expanded *ex vivo*. In principle, this might be due to selective resistance to apoptosis induced by the isolation procedure. After DAPI exclusion, virtually no ALDH^hi^ cells but part of the ALDH^lo^ population were Annexin V-positive (Figure 4A) suggesting they were in the early-mid apoptosis. The clonogenicity index was 0.27 ± 0.09% in ALDH^hi^ cells, 0% in ALDH^lo^ cells, and 0.03 ± 0.01% in the bulk (unsorted) population (Figure 4B). Freshly isolated ALDH^hi^ cells grew faster than the bulk population. Similarly, ALDH^hi^ outgrowth cells grew faster than ALDH^lo^ ones (Figure 4C). Expanded ALDH^hi^ cells lost ALDH activity over time (e.g., the frequency of ALDH^hi^ cells in the progeny of freshly isolated ALDH^hi^ cells after 13 culture-passages was 32%). These observations suggest that high ALDH activity may be required for the initial survival and growth of freshly isolated cells, but not in established cell cultures, reflecting cell adaptation to *in vitro* conditions.

### All-*trans* RA reverses DEAB-mediated cell growth suppression

To assess the effect of ALDH on cell growth, explant outgrowths were cultured with or without DEAB at varying concentrations. DEAB reduced proliferation in outgrowth cells in a dose-dependent manner (Figure 4D), as determined using an EdU incorporation assay. Because ALDH1 naturally synthesizes RA, we measured RA concentrations in ALDH^hi^ and ALDH^lo^ cells. Not surprisingly, we found higher RA levels in the former (Figure 4E). We then tested the ability of all*-trans* RA to replace ALDH in promoting cell growth. all*-trans* RA (10 μM) reversed cell growth suppression mediated by DEAB (Figure 4F). These results suggest that ALDH might promote atrial cell growth through RA, at least in part.

### ALDH^hi^ cells have higher propensity to produce mature cardiomyocytes than ALDH^lo^ cells

**Figure 5:**
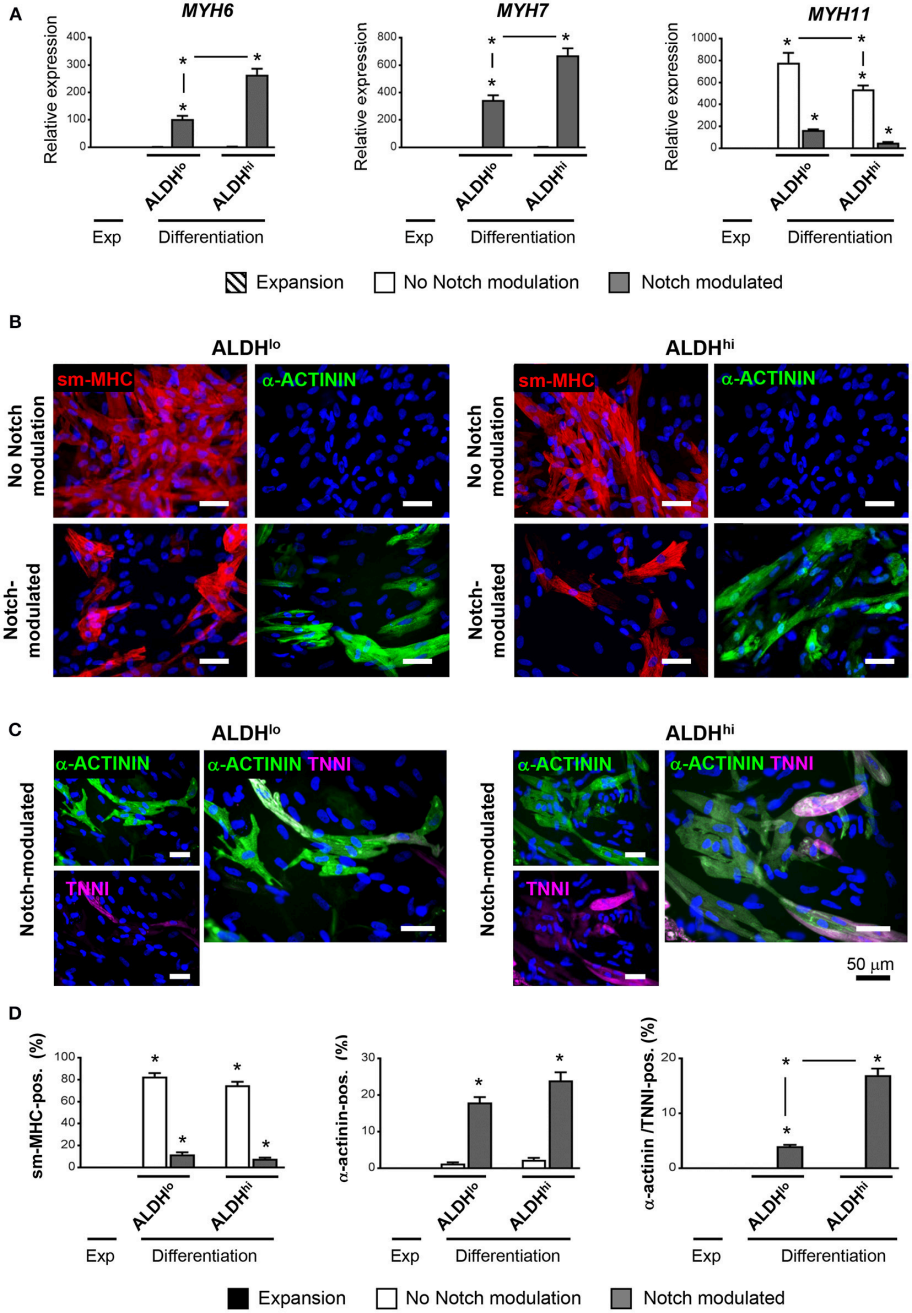
Cardiac differentiation of ALDH^hi^ and ALDH^lo^ atrial cells using a Notch modulation-based protocol. **(A)** real-time RT-PCR expression analyses of cardiac-specific (*Myh6, Myh7*) and smooth muscle-specific genes (*Myh11*) under expansion (Exp) and differentiation conditions, with or without Notch modulation. 2^−ΔCt^values are shown. One example of two independent experiments performed in triplicates is shown; *p < 0.05 vs. cells in expansion or between the indicated conditions. **(B)** Immunostaining of ALDH^hi^ and ALDH^lo^ cells for smooth musche-myosin heavy chain (smMHC) and cardiac-specific α-actinin, with or without Notch modulation. **(C)** Immunostaining of ALDH^hi^ and ALDH^lo^ cells for cardiac-specific α-actinin and troponin I (cTNNI) under Notch-modulated conditions. **(D)** Quantitative analyses of smMHC, α-actinin, and α-actinin/cTNNI positive cells; *p < 0.05 vs. cells in expansion (a minimum of 2000 cells in 30 different fields at 40x magnification were analyzed for quantification).

Having shown that the ALDH^hi^ population is enriched with stem/progenitor cells, we addressed its cardiomyogenic potential. Two differentiation protocols, with or without Notch modulation, were used. Under differentiation conditions with no Notch-modulation, both ALDH^hi^ and ALDH^lo^ cells expressed marked levels of MYH11 gene, while the cardiomyocyte markers MYH6 and MYH7 were not induced (Figure 5A). Both populations massively differentiated into smooth muscle cells, as illustrated by smMHC immunostaining; less than 2% of cells stained positive for cardiomyocyte-specific marker α-actinin (Figure 5B). By contrast, under Notch-modulated conditions both ALDH^lo^ and ALDH^hi^ cells expressed significant levels of cardiomyocyte-specific MYH6 and MYH7 (Figure 5A) and α-actinin (Figures 5B,D). Cardiac troponin-I (TNNI) was also identified in differentiated cells. Interestingly, under Notch-modulated conditions MYH6 and MYH7 gene expression was more pronounced in ALDH^hi^ than ALDH^lo^ cells. Similarly, the number of α-actinin/TNNI double positive cells derived from ALDH^hi^ cells was significantly higher than that derived from ALDH^lo^ cells (Figures 5C,D). These results indicated that the differentiated ALDH^hi^ cells were more terminally differentiated than ALDH^lo^ cells, consistent with a higher propensity of ALDH^hi^ cells to differentiate into cardiomyocytes.

### ALDH1A3 is the most highly expressed ALDH isoform in atrial cells

**Figure 6:**
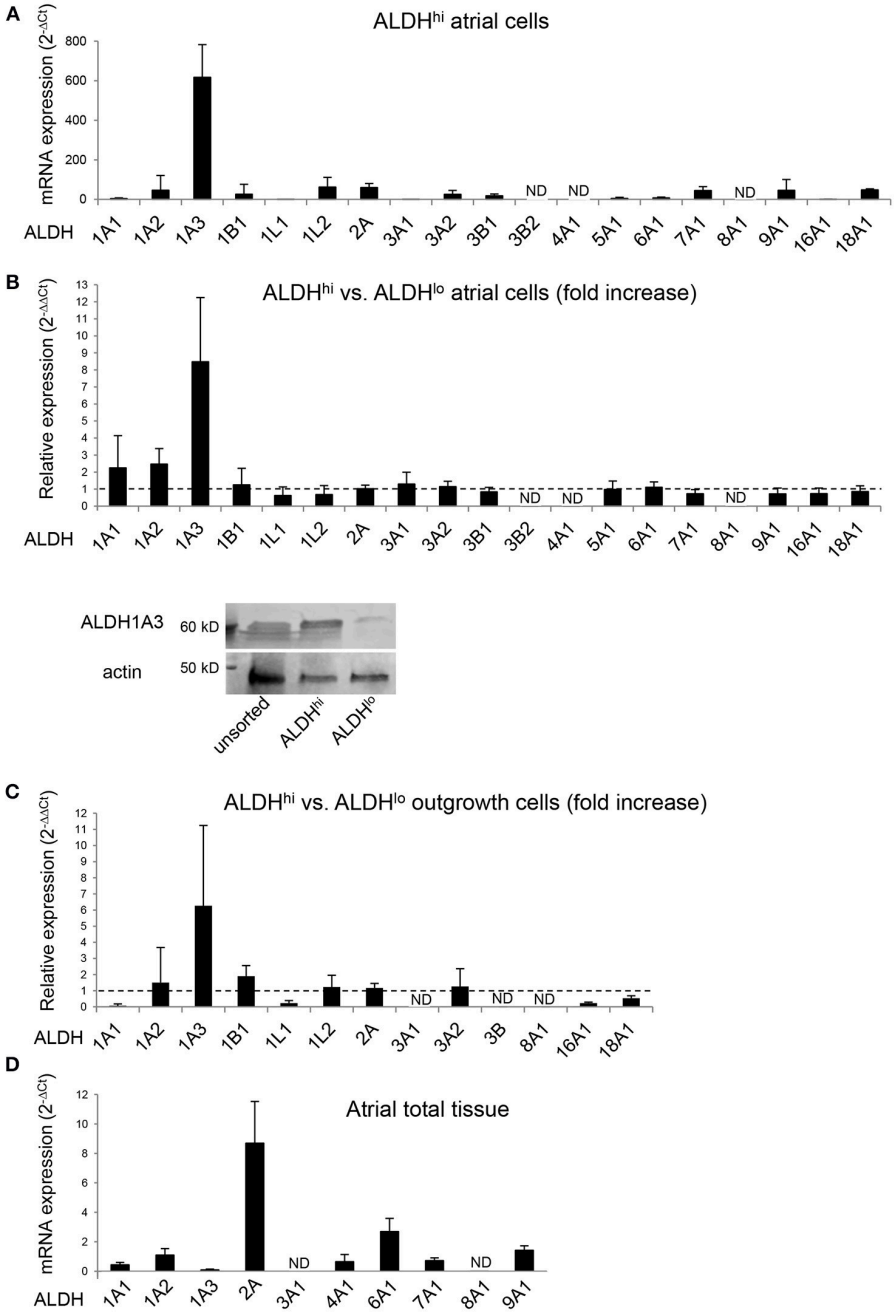
RT-PCR analysis of *ALDH* isoform gene expression in different populations. **(A)** ALDH^hi^ cells isolated using enzymatic techniques. Upper panel: ALDH isoform expression (2^−ΔCt^ values; *n* = 4 patients). Lower panel: Relative expression in ALDH^hi^ vs. ALDH^lo^ cells (2^−ΔΔCt^ values; *n* = 4). **(B)** Western blot showing ALDH1A3 protein expression in unsorted, ALDH^hi^ and ALDH^lo^ atrial cells. **(C)** Outgrowth cells. Relative expression in ALDH^hi^ vs. ALDH^lo^ cells is shown (*n* = 3). **(D)** Total atrial tissue (2^−ΔCt^ values; *n* = 3). ND, not determined (2^−ΔΔCt^ values that could not be calculated).

The specific ALDH isoforms expressed in ALDH^hi^ atrial cells have not been identified previously. We therefore analyzed the ALDH isoform expression profiles of ALDH^hi^ and ALDH^lo^ cells. ALDH1A3 was the most highly expressed isoform in freshly isolated ALDH^hi^ cells, as well as the most highly overexpressed isoform in ALDH^hi^ relative to ALDH^lo^ cells (≈8-fold increase; Figure 6A). ALDH1A3 protein levels in ALDH^hi^ cells were higher than in ALDH^lo^ cells, as evidenced by Western blot analysis (Figure 6B). Similar findings were observed in outgrowth cells (≈6-fold increase in ALDH1A3 mRNA levels in ALDH^hi^ vs. ALDH^lo^ cells; Figure 6C). For comparison, a subset of 10 ALDH isoforms selected based on published data (40) were measured in atrial total tissue, and ALDH2 was found to be the most highly expressed isoform (Figure 6D). Thus, ALDH1A3 is the key isoform expressed in ALDH^hi^ atrial cells.

### ALDH1A3 siRNA reduces ALDH activity and *in vitro* cell proliferation

**Figure 7:**
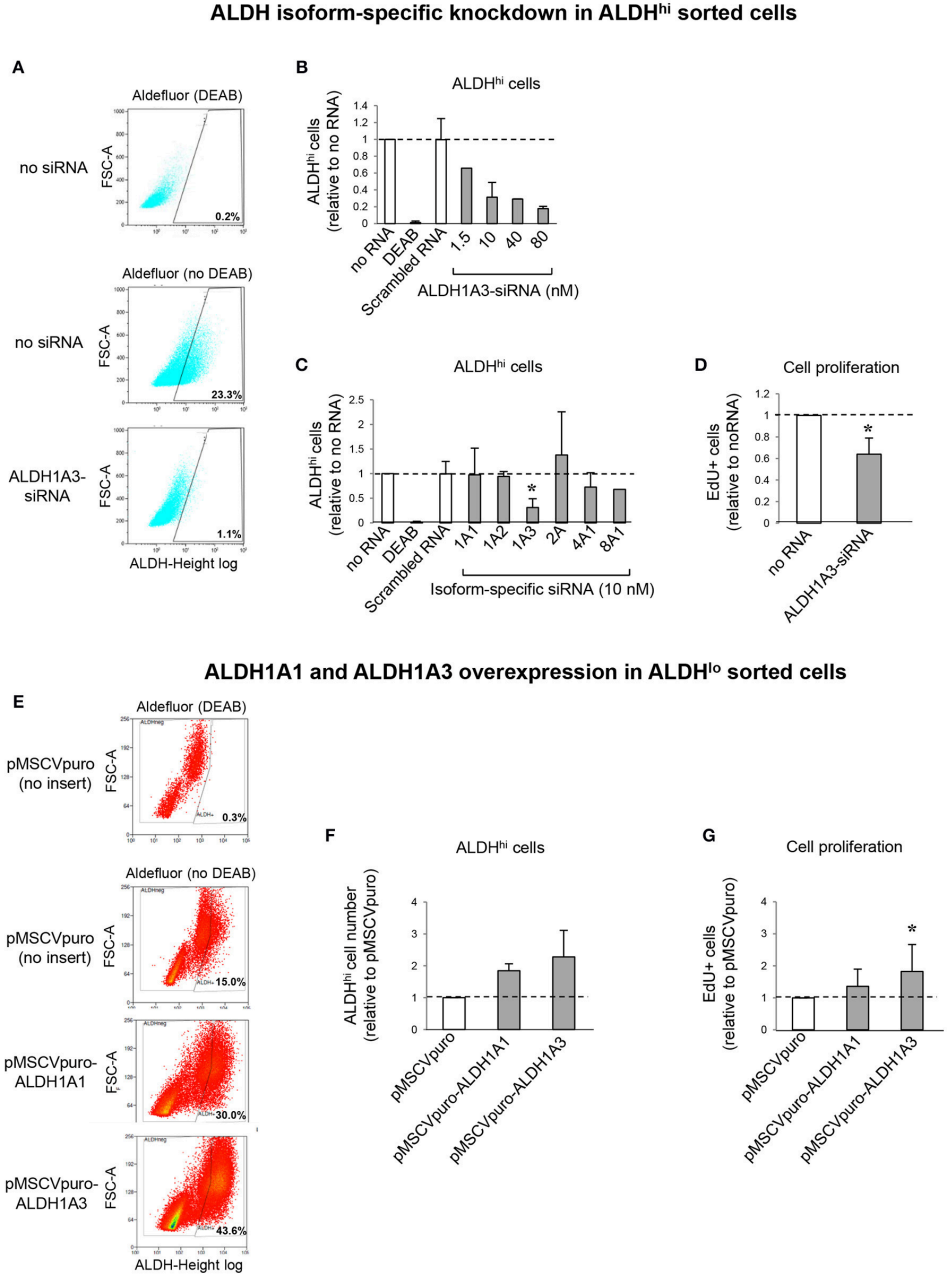
ALDH isoform-specific loss- and gain-of-function studies. **(A)** Flow cytometry plots of ALDH^hi^ sorted, Aldefluor-reacted cells (with or w/o DEAB) after siALDH1A3 transfection or mock transfection (no siRNA). **(B)** siALDH1A3 dose-response study. ALDH^hi^ cell numbers after siALDH1A3 transfection using the indicated concentrations relative no siRNA. **(C)** ALDH^hi^ cell numbers after siALDH-isoform-specific transfection (10 nM) relative to no siRNA). **(D)** EdU^+^ cell numbers (%) after siALDH1A3 transfection (10 nM) relative no siRNA. **(E)** Flow cytometry plots of ALDH^lo^ sorted, Aldefluor-reacted cells (with or w/o DEAB) transduced with pMSCVpuro-ALDH1A1 or pMSCVpuro-ALDH1A3 retroviral vectors, or control vector with no insert (pMSCVpuro). **(F)** ALDH^hi^ cell numbers after transduction with pMSCVpuro-ALDH1A1 or pMSCVpuro-ALDH1A3 relative to control vector. **(G)** EdU^+^ cell numbers (%) after transduction with pMSCVpuro-ALDH1A1 or pMSCVpuro-ALDH1A3 relative to control vector; *p < 0.05.

To address the contributions of individual ALDH isoforms to ALDH activity in ALDH^hi^ cells, we examined whether altering their expression levels would affect ALDH activity. Isoform-specific siRNAs were transfected into ALDH^hi^ sorted cells, which were then analyzed using the Aldefluor assay. siALDH1A3 reduced the percentage of ALDH^hi^ cells in a dose-dependent manner up to ≈90% (Figures 7A,B). On the other hand, siALDH1A1, siALDH1A2, siALDH2A, siALDH4A1, and siALDH8A1 (10 nM) did not significantly affect percentages of ALDH^hi^ cells (Figure 7C). To address the role of ALDH1A3 in cell proliferation, siALDH1A3 (10 nM) was transfected into ALDH^hi^ sorted cells and proliferation was assessed using EdU incorporation kit. siALDH1A3 reduced the number of EdU^+^ cells by ≈40% (Figure 7D).

### ALDH1A3 overexpression increases *in vitro* cell proliferation

Having shown that ALDH1A3 knockdown inhibits ALDH^hi^ cell proliferation, we overexpressed this isoform in ALDH^lo^ cells. Retrovirus-mediated ALDH1A3 gene transduction into ALDH^lo^ sorted cells enhanced both ALDH activity (Figures 7E,F) and cell proliferation (Figure 7G) compared with cells infected with control retrovirus with no insert. ALDH1A1 gene transduction also induced a minor increase in Aldefluor-positive cells. Collectively, these results indicate that ALDH1A3 promotes *in vitro* proliferation of atrial-derived cells.

## Discussion

We and others have shown previously that high ALDH activity defines a human cardiac atrial appendage cell population enriched with stem and progenitor cells that have cardiomyogenic potential (33, 35). ALDH^hi^ activity coincides with primitive surface marker (CD34^+^) as well as mesenchymal and endothelial progenitor marker expression. Regarding the co-expression of CD34 and CD31, CD34^+^ endothelial progenitor-like cells have been described previously (41). Interestingly, the ALDH^hi^ population is significantly enriched with VCAM1^+^ cells. Moreover, ALDH^hi^ cells differentially express both pluripotency genes and markers for more established cardiac lineages including Nkx2.5, consistent with them being enriched with both primitive cells and cells committed to a cardiac fate. VCAM1^+^Nkx2.5^+^ cells have been identified previously as a discrete lineage of myocardial committed cells during early human cardiovascular development (42). These findings raise the question as to whether the VCAM1^+^Nkx2.5^+^ subset of the adult ALDH^hi^ atrial population consists of myocardial committed progenitor cells. These results suggest that ALDH^hi^ atrial cells may be enriched with cells that have cardiomyogenic potential. To address this aspect, we used a novel differentiation protocol based on Notch modulation (38). Compared with ALDH^lo^ cells, ALDH^hi^ cells produced higher numbers of mature cardiomyocytes, as evidenced by higher expression levels of cardiac specific genes (MYH6, MYH7) and higher frequencies of cardiac-specific α-actinin/troponin I double-positive cells. These results indicate that the ALDH^hi^ population is enriched with cardiac progenitor cells. *In vivo* lineage tracing studies addressing the role of ALDH^hi^ cardiac cells are still needed.

Freshly isolated ALDH^hi^ cells, but not ALDH^lo^ cells, include a subset of clonogenic cells and can be expanded. Annexin V-staining suggests that ALDH^hi^ cells may be more apoptosis-resistant than ALDH^lo^ ones. Higher apoptosis-resistance of ALDH^hi^ vs. ALDH^lo^ cells has been reported in various cancer stem cells (43–45). Silencing ALDH1A by siRNA or shRNA in human melanoma cancer stem cells led to cell cycle arrest, apoptosis and decreased cell viability *in vitro* and reduced tumorigenesis *in vivo* (45). In our study, the theoretical possibility that low ALDH activity in a majority of freshly isolated cells is a result of apoptosis, rather than its cause, cannot be ruled out. Nevertheless, DAPI/Annexin-V double-negative ALDH^lo^ cells that were not in the early-mid apoptosis still failed to grow *in vitro*, suggesting that high ALDH activity might be required in the initial cell culture stages. On the other hand, both the ALDH^lo^ subset of the ALDH^hi^ progeny (i.e., ALDH^hi^ cells having lost their ALDH activity over time *in vitro*) and ALDH^lo^ outgrowth cells could be expanded, albeit with slower growth kinetics compared with ALDH^hi^ cells. This observation suggests that high ALDH activity may not be required in established cell cultures possibly due to cell adaptation mechanisms.

ALDH isoform expression analysis identified ALDH1A3 as both the most highly expressed isoform in ALDH^hi^ atrial cells and the main differentially expressed isoform in ALDH^hi^ vs. ALDH^lo^ cells. Similar results were found in both freshly isolated and outgrowth cells. Moreover, ALDH^hi^ cells showed higher ALDH1A3 protein levels than ALDH^lo^ cells. siALDH1A3 transfected into ALDH^hi^ cells reduced the number of cells that remained ALDH^hi^ by ≈90%, whereas knocking down ALDH1A1, ALDH1A2, ALDH2A, ALDH4A1, or ALDH8A1 did not significantly affect it. These findings establish ALDH1A3 as the key isoform responsible for ALDH activity in ALDH^hi^ cells. siALDH1A3 transfected into ALDH^hi^ cells reduced their proliferation. Specific ALDH inhibition by DEAB likewise suppressed cell proliferation. Conversely, retrovirus-mediated ALDH1A3 gene transduction into ALDH^lo^ cells promoted it. ALDH1A1 overexpression induced a similar albeit weaker effect, suggesting that exogenous ALDH1A1 may replace the naturally expressed ALDH1A3 isoform in this respect.

ALDH1A is the rate-limiting factor in the production of RA (16–18). We found higher RA levels in ALDH^hi^ atrial cells than in ALDH^lo^ cells. Moreover, the RA receptor agonist, all*-trans* RA, replaced ALDH in promoting atrial cell growth in the presence of DEAB. In breast cancer cell lines, ALDH1A3 influenced cancer progression via differential RA signaling (44). In human melanoma cancer stem cells, ALDH1A contributed to the stemness through RA-dependent and -independent pathways (45). In HSCs, all*-trans* RA reversed DEAB-mediated effects on cell expansion and differentiation, suggesting that the ability of ALDH to generate RA is important in determining HSC fate (13). Of note, the catalytic activity of ALDH1A3 for all*-trans* retinal may be 10-fold higher than that of ALDH1A1 (46). Moreover, ALDH1A3 is transcriptionally activated by all*-trans* RA in human epidermal keratinocytes, which raises the theoretical possibility of a positive feedback loop between ALDH1A3 and RA in some cell types (47). Our results are consistent with, but do not firmly establish, RA-dependent ALDH effects on atrial cell growth.

Functional roles of ALDH1A3 in stem/progenitor cells are poorly understood. ALDH1A3 promotes neural progenitor cell self-renewal through the regulation of ALDH1A3 pre-mRNA 3′-end processing by RNA-binding protein Sam68 (Khdrbs1). Decreases in ALDH1A3 expression and activity resulted in decreased clonogenicity and reduced the pool of proliferating neural progenitor cells (48). Several studies showed that ALDH1A3 is a cancer stem cell gene. ALDH1A3 expression was detected in 78% of small-sized Ki-67^+^ proliferating progenitor cells in the human breast cancer cell line HCC1937 (43). Most recently, ALDH1A3 was shown to affect colon cancer *in vitro* proliferation and invasion depending on CXCR4 status (49). In neuroblastoma cell lines expressing high ALDH1A3 levels, ALDH1A3 knockout via CRISPR/Cas9 gene editing reduced clonogenicity and mediated cell type-dependent inhibition of self-renewal properties of tumor inducing cells (50). Collectively, these findings indicate that ALDH1A3 play functional roles in both normal and cancer stem/progenitor cells.

In conclusion, our results suggest that the ALDH^hi^ population isolated from human cardiac atrial appendages may be enriched with stem/progenitor cells showing cardiomyogenic potential, and that ALDH1A3 is the key ALDH isoform responsible for ALDH activity in these cells, affecting their *in vitro* proliferation.

## Author contributions

Substantial contributions to the conception or design of the work (GV and SP) or the acquisition, analysis, or interpretation of data for the work (SP, IP, LB, EC, GM, PM, TP, GV), Drafting the work or revising it critically for important intellectual content (SP, IP, GV), Final approval of the version to be published, Agreement to be accountable for all aspects of the work in ensuring that questions related to the accuracy or integrity of any part of the work are appropriately investigated and resolved (SP, IP, LB, EC, GM, PM, TP, GV).

### Conflict of interest statement

The authors declare that the research was conducted in the absence of any commercial or financial relationships that could be construed as a potential conflict of interest.

## References

[1] Sidney LE Branch MJ Dunphy SE Dua HS Hopkinson A. Concise review: evidence for CD34 as a common marker for diverse progenitors. Stem Cells (2014) 32:1380–9. 10.1002/stem.1661 24497003 PMC4260088

[2] Tateishi K Ashihara E Takehara N Nomura T Honsho S Nakagami T. Clonally amplified cardiac stem cells are regulated by Sca-1 signaling for efficient cardiovascular regeneration. J Cell Sci. (2007) 120:1791–800. 10.1242/jcs.006122 17502484

[3] Beltrami AP Barlucchi L Torella D Baker M Limana F Chimenti S. Adult cardiac stem cells are multipotent and support myocardial regeneration. Cell (2003) 114:763–76. 14505575 10.1016/s0092-8674(03)00687-1

[4] Vicinanza C Aquila I Scalise M Cristiano F Marino F Cianflone E. Adult cardiac stem cells are multipotent and robustly myogenic: c-kit expression is necessary but not sufficient for their identification. Cell Death Differ. (2017) 24:2101–16. 10.1038/cdd.2017.130 28800128 PMC5686347

[5] Cai J Weiss ML Rao MS. In search of “stemness”. Exp Hematol. (2004) 32:585–98. 10.1016/j.exphem.2004.03.013 15246154 PMC3279197

[6] Moreb JS. Aldehyde dehydrogenase as a marker for stem cells. Curr Stem Cell Res Ther. (2008) 3:237–46. 10.2174/157488808786734006 19075754

[7] Balber AE. Concise review: aldehyde dehydrogenase bright stem and progenitor cell populations from normal tissues: characteristics, activities, and emerging uses in regenerative medicine. Stem Cells (2011) 29:570–5. 10.1002/stem.613 21308868

[8] Xu X Chai S Wang P Zhang C Yang Y Wang K. Aldehyde dehydrogenases and cancer stem cells. Cancer Lett (2015) 369:50–7. 10.1016/j.canlet.2015.08.018 26319899

[9] Laird DJ De Tomaso AW Weissman IL. Stem cells are units of natural selection in a colonial ascidian. Cell (2005) 123:1351–60. Erratum in: Cell (2006) 124:647–8. 10.1016/j.cell.2005.10.026 16377573

[10] Jackson B Brocker C Thompson DC Black W Vasiliou K Nebert DW. Update on the aldehyde dehydrogenase gene (ALDH) superfamily. Hum Genomics (2011) 5:283–303. 10.1186/1479-7364-5-4-283 21712190 PMC3392178

[11] Marchitti SA Brocker C Stagos D Vasiliou V. Non-P450 aldehyde oxidizing enzymes: the aldehyde dehydrogenase superfamily. Expert Opin Drug Metab Toxicol. (2008) 4:697–720. 10.1517/17425255.4.6.697 18611112 PMC2658643

[12] Kastan MB Schlaffer E Russo JE Colvin OM Civin CI Hilton J Direct demonstration of elevated aldehyde dehydrogenase in human hematopoietic progenitor cells. Blood (1990) 74:1945–50. 2337669

[13] Chute JP Muramoto GG Whitesides J Colvin M Safi R Chao NJ. Inhibition of aldehyde dehydrogenase and retinoid signaling induces the expansion of human hematopoietic stem cells. Proc Natl Acad Sci USA (2006) 103:11707–12. 10.1073/pnas.0603806103 16857736 PMC1544234

[14] Corti S Locatelli F Papadimitriou D Donadoni C Del Bo R Crimi M. Transplanted ALDHhiSSClo neural stem cells generate motor neurons and delay disease progression of nmd mice, an animal model of SMARD1. Hum Mol Genet (2006) 15:167–87. 10.1093/hmg/ddi446 16339214

[15] Hilton J Role of aldehyde dehydrogenase in cyclophosphamide resistant L1210 leukemia. Cancer Res. (1984) 445:156. 6488175

[16] Zhao D McCaffery P Ivins KJ Neve RL Hogan P Chin WW. Molecular identification of a major retinoic-acid-synthesizing enzyme, a retinaldehyde-specific dehydrogenase. Eur J Biochem (1996) 240:15–22. 8797830 10.1111/j.1432-1033.1996.0015h.x

[17] Elizondo G Corchero J Sterneck E Gonzalez FJ. Feedback inhibition of the retinaldehyde dehydrogenase gene ALDH1 by retinoic acid through retinoic acid receptor alpha and CCAAT/enhancer-binding protein beta. J Biolog Chem. (2000) 275:39747–353. 10.1074/jbc.M004987200 10995752

[18] Black W Vasiliou V The aldehyde dehydrogenase gene superfamily resource center. Hum Genomics (2009) 136:e142 10.1186/1479-7364-4-2-136 PMC3525204 20038501

[19] Collins SJ. Retinoic acid receptors, hematopoiesis and leukemogenesis. Curr Opin Hematol. (2008) 15:346–51. 10.1097/MOH.0b013e3283007edf 18536573

[20] Marcato P Dean CA Giacomantonio CA Lee PW. Aldehyde dehydrogenase: its role as a cancer stem cell marker comes down to the specific isoform. Cell Cycle (2011) 10:1378–84. 10.4161/cc.10.9.15486 21552008

[21] Ginestier C Hur MH Charafe-Jauffret E Monville F Dutcher J Brown M. ALDH1 is a marker of normal and malignant human mammary stem cells and a predictor of poor clinical outcome. Cell Stem Cell (2007) 1:555–67. 10.1016/j.stem.2007.08.014 18371393 PMC2423808

[22] Burger PE Gupta R Xiong X Ontiveros CS Salm SN Moscatelli D. High aldehyde dehydrogenase activity: a novel functional marker of murine prostate stem/progenitor cells. Stem Cells (2009) 27:2220–8. 10.1002/stem.135 19544409 PMC2887284

[23] Marcato P Dean C Pan D Araslanova R Gillis M Joshi M. Aldehyde dehydrogenase activity of breast cancer stem cells is primarily due to isoform ALDH1A3 and its expression is predictive of metastasis. Stem Cells (2011) 29:32–45. 10.1002/stem.563 21280157

[24] Singh S Arcaroli J Chen Y Thompson DC Messersmith W Jimeno A. ALDH1B1 is crucial for colon tumorigenesis by modulating Wnt/beta-Catenin, notch and PI3K/Akt signaling pathways. PLoS ONE (2015) 10:e0121648. 10.1371/journal.pone.0121648 25950950 PMC4423958

[25] Yan J De Melo J Cutz JC Aziz T Tang D. Aldehyde dehydrogenase 3A1 associates with prostate tumorigenesis. Br J Cancer (2014) 110:2593–603. 10.1038/bjc.2014.201 24762960 PMC4021532

[26] Storms RW Trujillo AP Springer JB Shah L Colvin OM Luderman S. Isolation of primitive human hematopoietic progenitors on the basis of aldehyde dehydrogenase activity. Proc Natl Acad Sci USA. (1999) 96:9118–23. 10430905 10.1073/pnas.96.16.9118 PMC17742

[27] Gunduz E Demirel G Bal C Gulbas Z. Evaluation of mobilized peripheral stem cells according to CD34 and aldehyde dehydrogenase expression and effect of SSC(lo) ALDH(br) cells on hematopoietic recovery. Cytotherapy (2010) 12:1006–12. 10.3109/14653249.2010.509393 20735165

[28] Shoulars K Noldner P Troy JD Cheatham L Parrish A Gentry T. Development and validation of a rapid, aldehyde dehydrogenase bright-based cord blood potency assay. Blood (2016) 127:2346–54. 10.1182/blood-2015-08-666990 26968535 PMC4865591

[29] Capoccia BJ Robson DL Levac KD Maxwell DJ Hohm SA Neelamkavil MJ. Revascularization of ischemic limbs after transplantation of human bone marrow cells with high aldehyde dehydrogenase activity. Blood (2009) 113:5340–51. 10.1182/blood-2008-04-154567 19324906 PMC2686196

[30] Sondergaard CS Hess DA Maxwell DJ Weinheimer C Rosová I Creer MH. Human cord blood progenitors with high aldehyde dehydrogenase activity improve vascular density in a model of acute myocardial infarction. J Transl Med. (2010) 8:24. 10.1186/1479-5876-8-24 20214792 PMC2846892

[31] Perin EC Murphy MP March KL Bolli R Loughran J Yang PC Cardiovascular cell therapy research network (CCTRN). Evaluation of cell therapy on exercise performance and limb perfusion in peripheral artery disease: The CCTRN PACE Trial (Patients with intermittent claudication injected with ALDH bright cells). Circulation (2017) 135:1417–28. 10.1161/CIRCULATIONAHA.116.025707 28209728 PMC5388585

[32] Perin EC Silva GV Zheng Y Gahremanpour A Canales J Patel D. Randomized, double-blind pilot study of transendocardial injection of autologous aldehyde dehydrogenase-bright stem cells in patients with ischemic heart failure. Am Heart J (2012) 163:415–21. 10.1016/j.ahj.2011.11.020 22424012

[33] Spicher A Meinhardt A Roehrich ME Vassalli G Phenotypic characterization of murine and human cardiac-resident progenitor cells isolated on basis of aldehyde-dehydrogenase activity. Circulation (2007) 116:II−167–II−168.

[34] Roehrich ME Spicher A Milano G Vassalli G. Characterization of cardiac-resident progenitor cells expressing high aldehyde dehydrogenase activity. Biomed Res Int. (2013) 2013:503047. 10.1155/2013/503047 23484127 PMC3581094

[35] Koninckx R Daniëls A Windmolders S Mees U Macianskiene R Mubagwa K. The cardiac atrial appendage stem cell: a new and promising candidate for myocardial repair. Cardiovasc Res. (2013) 97:413–23. 10.1093/cvr/cvs427 23257022

[36] Morgan CA Parajuli B Buchman CD Dria K Hurley TD. N,N-diethylaminobenzaldehyde (DEAB) as a substrate and mechanism-based inhibitor for human ALDH isoenzymes. Chem Biol Interact. (2015) 234:18–28. 10.1016/j.cbi.2014.12.008 25512087 PMC4414715

[37] Messina E De Angelis L Frati G Morrone S Chimenti S Fiordaliso F. Isolation and expansion of adult cardiac stem cells from human and murine heart. Circ Res. (2004) 95:911–21. 10.1161/01.RES.0000147315.71699.51 15472116

[38] Plaisance I Perruchoud S Fernandez-Tenorio M Gonzales C Ounzain S Ruchat P. Cardiomyocyte lineage specification in adult human cardiac precursor cells via modulation of enhancer-associated long noncoding RNA expression. J Am Coll Cardiol Basic Trans Sci. (2016) 1:472–93. 10.1016/j.jacbts.2016.06.008 29707678 PMC5916868

[39] Makkar RR Smith RR Cheng K Malliaras K Thomson LE Berman D. Intracoronary cardiosphere-derived cells for heart regeneration after myocardial infarction (CADUCEUS): a prospective, randomised phase 1 trial. Lancet (2012) 379:895–904. 10.1016/S0140-6736(12)60195-0 22336189 PMC4326004

[40] Alnouti Y Klaassen CD. Tissue distribution, ontogeny, and regulation of aldehyde dehydrogenase (Aldh) enzymes mRNA by prototypical microsomal enzyme inducers in mice. Tox Sci. (2008) 101:51–64. 10.1093/toxsci/kfm280 17998271

[41] Ferreras C Cole CL Urban K Jayson GC Avizienyte E. Segregation of late outgrowth endothelial cells into functional endothelial CD34- and progenitor-like CD34+ cell populations. Angiogenesis (2015) 18:47–68. 10.1007/s10456-014-9446-1 25269667

[42] Skelton RJP Costa M Anderson DJ Bruveris F Finnin BW Koutsis K. SIRPA, VCAM1 and CD34 identify discrete lineages during early human cardiovascular development. Stem Cell Res. (2014) 13:172–9. 10.1016/j.scr.2014.04.016 24968096

[43] Kashii-Magaribuchi K Takeuchi R Haisa Y Sakamoto A Itoh A Izawa Y. Induced expression of cancer stem cell markers ALDH1A3 and Sox-2 in hierarchical reconstitution of apoptosis-resistant human breast cancer cells. Acta Histochem Cytochem. (2016) 49:149–58. 10.1267/ahc.16031 27917009 PMC5130344

[44] Marcato P Dean CA Liu RZ Coyle KM Bydoun M Wallace M. Aldehyde dehydrogenase 1A3 influences breast cancer progression via differential retinoic acid signaling. Mol Oncol. (2015) 9:17–31. 10.1016/j.molonc.2014.07.010 25106087 PMC5528683

[45] Luo Y Dallaglio K Chenc Y Robinson WA Robinson SE McCarter MD. ALDH1A isozymes are markers of human melanoma stem cells and potential therapeutic targets. Stem Cells (2012) 30:2100–13. 10.1002/stem.1193 22887839 PMC3448863

[46] Sima A Parisotto M Mader S Bhat PV. Kinetic characterization of recombinant mouse retinal dehydrogenase types 3 and 4 for retinal substrates. Biochim Biophys Acta (2009) 1790:1660–4. 10.1016/j.bbagen.2009.09.004 19766701

[47] Koenig U Amatschek S Mildner M Eckhart L Tschachler E. Aldehyde dehydrogenase 1A3 is transcriptionally activated by all-trans-retinoic acid in human epidermal keratinocytes. Biochem Biophys Res Commun. (2010) 400:207–11. 10.1016/j.bbrc.2010.08.035 20709019

[48] La Rosa P Bielli P Compagnucci C Cesari E Volpe E Farioli Vecchioli S Sam68 promotes self-renewal and glycolytic metabolism in mouse neural progenitor cells by modulating Aldh1a3 pre-mRNA 3′-end processing. Elife (2016) 5:e20750 10.7554/eLife.20750 27845622 PMC5122457

[49] Feng H Liu Y Bian X Zhou F Liu Y. ALDH1A3 affects colon cancer *in vitro* proliferation and invasion depending on CXCR4 status. Br Cancer (2018) 118:224–32. 10.1038/bjc.2017.363 29235568 PMC5785736

[50] Flahaut M Jauquier N Chevalier N Nardou K Balmas Bourloud K Joseph JM. Aldehyde dehydrogenase activity plays a key role in the aggressive phenotype of neuroblastoma. BMC Cancer (2016) 16:781. 10.1186/s12885-016-2820-1 27724856 PMC5057398

