# Supplementary material for: ALDH1A3 Is the Key Isoform That Contributes to Aldehyde Dehydrogenase Activity and Affects in Vitro Proliferation in Cardiac Atrial Appendage Progenitor Cells
Source: Front Cardiovasc Med. 2018 Jul 24;5:90. doi: 10.3389/fcvm.2018.00090 (PMC6066537; doi:10.3389/fcvm.2018.00090)
Supplement: Supplementary file 1 [file Table_1.DOCX]

**Suppl. Table 1**

Antibodies used in flow cytometry studies (10 μL/10^6^ cells)

| Antibody | from: |
| --- | --- |
| mouse monoclonal anti-human CD13 (APC) | eBioscience |
| mouse monoclonal anti-human CD31 (Alexafluor 647) | Biolegend |
| mouse monoclonal anti-human CD34 (PE) | Beckman Coulter |
| mouse monoclonal anti-human CD38 (APC) | eBioscience |
| mouse monoclonal anti-human CD44 (APC) | eBioscience |
| mouse monoclonal anti-human CD45 (APC) | eBioscience |
| mouse monoclonal anti-human CD49a (Alexafluor 647) | Serotec |
| mouse monoclonal anti-human CD49d (APC) | Biolegend |
| mouse monoclonal anti-human CD71 (Alexafluor 647) | Serotec |
| mouse monoclonal anti-human CD90 (PE) | Biolegend |
| mouse monoclonal anti-human CD105 (PE) | Biolegend |
| mouse monoclonal anti-human CD106 (APC) | eBioscience |
| mouse monoclonal anti-human CD117 (APC) | eBioscience |
| mouse monoclonal anti-human CD133 (APC) | Biolegend |
