# Supplementary material for: ALDH1A3 Is the Key Isoform That Contributes to Aldehyde Dehydrogenase Activity and Affects in Vitro Proliferation in Cardiac Atrial Appendage Progenitor Cells
Source: Front Cardiovasc Med. 2018 Jul 24;5:90. doi: 10.3389/fcvm.2018.00090 (PMC6066537; doi:10.3389/fcvm.2018.00090)
Supplement: Supplementary file 2 [file Table_2.DOCX]

**Suppl. Table 2**

Antibodies used for immunocytochemistry

|  |  |  |
| --- | --- | --- |
| mouse monolyclonal anti-NKX2.5 | R&D systems | 1:100 |
| rabbit polyclonal anti-MEF2C | Abcam | 1:200 |
| rabbit polyclonal anti-GATA4 | Abcam | 1:200 |
| mouse monolyclonal anti sarcomeric α-actinin | Sigma | 1:600 |
| rabbit polyclonal anti smooth muscle myosin heavy chain (sm-MHC) | Biomedical Technologies Inc | 1:200 |
| rabbit polyclonal anti troponinI (TNNI) | Abcam | 1:200 |
| Alexa Fluor 488 conjugated secondary antibody | Molecular Probes | 1:200 |
| Alexa Fluor 594 conjugated secondary antibody | Molecular Probes | 1:200 |
| Alexa Fluor 647 conjugated secondary antibody | Molecular Probes | 1:200 |
