# Supplementary material for: ALDH1A3 Is the Key Isoform That Contributes to Aldehyde Dehydrogenase Activity and Affects in Vitro Proliferation in Cardiac Atrial Appendage Progenitor Cells
Source: Front Cardiovasc Med. 2018 Jul 24;5:90. doi: 10.3389/fcvm.2018.00090 (PMC6066537; doi:10.3389/fcvm.2018.00090)
Supplement: Supplementary file 3 [file Table_3.DOCX]

**Suppl. Table 3**

Primer sequences used in PCR analyses of ALDH isoform expression

|  | Foward primer sequence | Reverse primer sequence |
| --- | --- | --- |
| ALDH1A1 | GCACGCCAGACTTACCTGTC | CCACTCACTGAATCATGCCA |
| ALDH1A2 | GGCAGCAATAGCTTCTCACA | TTCTTCCAGCTGCTTCTTGG |
| ALDH1A3 | ATCAACTGCTACAACGCCCT | TATTCGGCCAAAGCGTATTC |
| ALDH1B1 | ACCAGAACCCAAGCGTGAT | CTGCTGCCGAGGAGTAGC |
| ALDH1L1 | CTGCGGCTCACAGAAGTCTA | CCAAACAGGCTCTGTCCAAT |
| ALDH1L2 | GGTGAAAGTGGCAGAGACCAT | CTTCAACCAGCCTGGCAACATC |
| ALDH2 | TCTTGATACGGGCAAAGGAC | TCCTGGTCCCCTTCAGTAC |
| ALDH3A1 | GCAGACCTGCACAAGAATGA | TGTAGAGCTCGTCCTGCTGA |
| ALDH3A2 | TGCACTTCACGCTCAACTCT | GGGACGCTGATAGAAAAAG |
| ALDH3B1 | CACCATGGAATCACTGCATC | GCCAGGCTGATCTTGAACTC |
| ALDH3B2 | AGGCAGCATGTCCAAGAAAG | AGTGTGTCCTCGAAGGGGT |
| ALDH4A1 | GTACGGTGGCCAGAAGTGTT | TCTTGATACGGGCAAAGGAC |
| ALDH5A1 | AGGCTGGGATTCCTTCAGGTGT | CAGAGTTTGCTGCGTGGTGCA |
| ALDH6A1 | GGCTCTTTCAACAGCAGTCC | ATGGAAGCTCCCTCCTTTGT |
| ALDH7A1 | CGAGCCAATAGCAAGAGTCC | CTTCACCCACACCTTCCACT |
| ALDH8A1 | TGGTGAGCATAGGTGCTCTG | GTTATCACCGTGGGAAGCAT |
| ALDH9A1 | CACTCATCAACCGACCACAC | GGACATAACAGGCCCAAAGA |
| ALDH16A1 | CTGCTCCACTACCATGCAATCC | GCAGGGCAAATCCTCCACATCA |
| ALDH18A1 | CTGAGTATGGGGACCTGGAA | GCGGTAACCATCAGAAAAGC |
